# Supplementary material for: An open-source framework for large-scale, flexible evaluation of biomedical text mining systems
Source: J Biomed Discov Collab. 2008 Jan 29;3:1. doi: 10.1186/1747-5333-3-1 (PMC2276192; doi:10.1186/1747-5333-3-1)
Supplement: Additional file 1 — Evaluation platform source code. The additional file contains the software evaluation framework discussed in this paper. The most current release can be downloaded from SourceForge.net [1]. See the accompanying README file for instructions on installation and use. Also included in the distribution is a collection of UIMA wrappers for some commonly used BioNLP tools and annotated corpora. [file 1747-5333-3-1-S1.zip › bionlp_uima_repository-1.0.1/docs/api/edu/uchsc/ccp/util/nlp/annotation/comparison/SpanComparator.html]

SpanComparator (Center for Computational Pharmacology Util/UIMA API)


|  |  |  |
| --- | --- | --- |
| **Overview** \| **Package** \| **Class** \| **Tree** \| **Deprecated** \| **Index** \| **Help** | |  |
| **PREV CLASS**   **NEXT CLASS** | **FRAMES**    **NO FRAMES**     **All Classes** |  |
| SUMMARY: NESTED \| FIELD \| CONSTR \| METHOD | DETAIL: FIELD \| CONSTR \| METHOD |  |


---


## edu.uchsc.ccp.util.nlp.annotation.comparison Class SpanComparator

```
java.lang.Object
  edu.uchsc.ccp.util.nlp.annotation.comparison.SpanComparator
```

**All Implemented Interfaces:**: Comparator<Span>

**Direct Known Subclasses:**: IgnoreSpanComparator, SharedEndSpanComparator, SharedStartOrEndSpanComparator, SharedStartSpanComparator, SloppySpanComparator, StrictSpanComparator

---

``` public abstract class SpanComparator extends Object implements Comparator<Span> ```

---

**Constructor Summary**

`SpanComparator()`


| **Method Summary** | |
| --- | --- |
| `abstract  int` | `compare(List<Span> spanList1, List<Span> spanList2)`             This method will be overridden by subclasses of SpanComparator |
| `abstract  int` | `compare(Span span1, Span span2)`             This method will be overridden by subclasses of SpanComparator |
| `int` | `matches(List<Span> spanList1, List<Span> spanList2)`             Compares a list of spans. |
| `int` | `matches(Span span1, Span span2)`             Returns 0 if the spans are equal, -1 if span1 starts before span2, 1 if span1 starts after span2. |
| `int` | `overlaps(List<Span> spanList1, List<Span> spanList2)`             Returns 0 if the span lists overlap. |
| `int` | `overlaps(Span span1, Span span2)`             Returns 0 if span1 and span2 overlap. |
| `int` | `sharesSpanEnd(List<Span> spanList1, List<Span> spanList2)`             Returns 0 if span1 and span2 share the same end index. |
| `int` | `sharesSpanEnd(Span span1, Span span2)`             Returns 0 if span1 and span2 share the same end index. |
| `int` | `sharesSpanStart(List<Span> spanList1, List<Span> spanList2)`             Returns 0 if span1 and span2 share the same start index. |
| `int` | `sharesSpanStart(Span span1, Span span2)`             Returns 0 if span1 and span2 share the same start index. |
| `int` | `sharesSpanStartOrEnd(List<Span> spanList1, List<Span> spanList2)`             Returns 0 if span1 and span2 share the same start or end index (or both). |
| `int` | `sharesSpanStartOrEnd(Span span1, Span span2)`             Returns 0 if span1 and span2 share the same start or end index (or both). |

**Methods inherited from class java.lang.Object**

`clone, equals, finalize, getClass, hashCode, notify, notifyAll, toString, wait, wait, wait`

**Methods inherited from interface java.util.Comparator**

`equals`

**Constructor Detail**

### SpanComparator

```
public SpanComparator()
```


**Method Detail**

### compare

```
public abstract int compare(Span span1,
                            Span span2)
```

:   This method will be overridden by subclasses of SpanComparator

    :   **Specified by:**: `compare` in interface `Comparator<Span>`

---


### compare

```
public abstract int compare(List<Span> spanList1,
                            List<Span> spanList2)
```

:   This method will be overridden by subclasses of SpanComparator

    :   **Parameters:**: `spanList1` -: `spanList2` - **Returns:**

---


### matches

```
public int matches(Span span1,
                   Span span2)
```

:   Returns 0 if the spans are equal, -1 if span1 starts before span2, 1 if span1 starts after span2.   
    If the spans start at the same index, then -1 if span1 ends before span2 and 1 if span1 ends after span2

---


### matches

```
public int matches(List<Span> spanList1,
                   List<Span> spanList2)
```

:   Compares a list of spans. If the lists have identical contents, return 0. If spanList1 starts before spanList2, return -1, otherwise return 1.  
    Note: an empty list will also return 0.

---


### overlaps

```
public int overlaps(Span span1,
                    Span span2)
```

:   Returns 0 if span1 and span2 overlap. Otherwise, it returns -1 if span1 starts before span2, and 1 if span1 starts after span2

    :   **Parameters:**: `span1` -: `span2` - **Returns:**

---


### overlaps

```
public int overlaps(List<Span> spanList1,
                    List<Span> spanList2)
```

:   Returns 0 if the span lists overlap. This method requires piecemeal overlap. That is, a list containing [1,5] [15,20] overlaps with a list
    containing [3,6] but does not overlap with the list: [6,11] [22,25]. Otherwise, it returns -1 if span1 starts before span2, and 1 if span1
    starts after span2

    :   **Parameters:**: `span1` -: `span2` - **Returns:**

---


### sharesSpanStart

```
public int sharesSpanStart(Span span1,
                           Span span2)
```

:   Returns 0 if span1 and span2 share the same start index. Otherwise, it returns -1 if span1 starts before span2, and 1 if span1 starts after
    span2

    :   **Parameters:**: `span1` -: `span2` - **Returns:**

---


### sharesSpanStart

```
public int sharesSpanStart(List<Span> spanList1,
                           List<Span> spanList2)
```

:   Returns 0 if span1 and span2 share the same start index. Otherwise, it returns -1 if span1 starts before span2, and 1 if span1 starts after
    span2

    :   **Parameters:**: `span1` -: `span2` - **Returns:**

---


### sharesSpanEnd

```
public int sharesSpanEnd(Span span1,
                         Span span2)
```

:   Returns 0 if span1 and span2 share the same end index. Otherwise, it returns -1 if span1 starts before span2, and 1 if span1 starts after span2

    :   **Parameters:**: `span1` -: `span2` - **Returns:**

---


### sharesSpanEnd

```
public int sharesSpanEnd(List<Span> spanList1,
                         List<Span> spanList2)
```

:   Returns 0 if span1 and span2 share the same end index. Otherwise, it returns -1 if span1 starts before span2, and 1 if span1 starts after span2

    :   **Parameters:**: `span1` -: `span2` - **Returns:**

---


### sharesSpanStartOrEnd

```
public int sharesSpanStartOrEnd(Span span1,
                                Span span2)
```

:   Returns 0 if span1 and span2 share the same start or end index (or both). Otherwise, return -1 if span1 starts before span2, and 1 if span1
    starts after span2

    :   **Parameters:**: `span1` -: `span2` - **Returns:**

---


### sharesSpanStartOrEnd

```
public int sharesSpanStartOrEnd(List<Span> spanList1,
                                List<Span> spanList2)
```

:   Returns 0 if span1 and span2 share the same start or end index (or both). Otherwise, return -1 if span1 starts before span2, and 1 if span1
    starts after span2

    :   **Parameters:**: `span1` -: `span2` - **Returns:**


---


|  |  |  |
| --- | --- | --- |
| **Overview** \| **Package** \| **Class** \| **Tree** \| **Deprecated** \| **Index** \| **Help** | |  |
| **PREV CLASS**   **NEXT CLASS** | **FRAMES**    **NO FRAMES**     **All Classes** |  |
| SUMMARY: NESTED \| FIELD \| CONSTR \| METHOD | DETAIL: FIELD \| CONSTR \| METHOD |  |


---
